# How infectious diseases arrived in the colonial Americas

**DOI:** 10.7554/eLife.72791

**Published:** 2021-09-09

**Authors:** Ville N Pimenoff, Charlotte J Houldcroft

**Affiliations:** 1 Department of Laboratory Medicine, Karolinska Institutet Stockholm Sweden; 2 Department of Cultures, University of Helsinki Helsinki Finland; 3 Faculty of Medicine, University of Oulu Oulu Finland; 4 Cambridge Institute of Therapeutic Immunology and Infectious Disease, Department of Medicine, University of Cambridge School of Clinical Medicine Cambridge United Kingdom

**Keywords:** ancient viruses, Africa, paleovirology, b19v, hbv, paleogenomics, Human, Virus

## Abstract

Analysis of viral DNA from human remains suggests that the transatlantic slave trade may have introduced new pathogens that contributed to the devastating disease outbreaks in colonial Mexico.

**Related research article** Guzmán-Solís AA, Villa-Islas V, Bravo-López MJ, Sandoval-Velasco M, Wesp JK, Gómez-Valdés JA, Moreno-Cabrera ML, Meraz A, Solís-Pichardo G, Schaaf P, TenOever BR, Blanco-Melo D, Ávila Arcos MC. 2021. Ancient viral genomes reveal introduction of human pathogenic viruses into Mexico during the transatlantic slave trade. *eLife* **10**:e68612. doi: 10.7554/eLife.68612

When European colonisers arrived in the Americas in the 16th and 17th centuries, they brought with them multiple infectious diseases which led to massive outbreaks that killed millions of indigenous people. However, it is unclear which pathogens were responsible for these devastating epidemics that are generally referred to as the Cocoliztli, meaning ‘pest’ in the Nahuatl language (Acuna-Soto et al., 2004).

Most research in this area has focused on diseases that arrived in the Americas from Europe, and do not factor in another horrific part of European colonial history: the transatlantic slave trade that forcibly transported West Africans – who carried unique genotypes of the disease-causing pathogens – to the Americas (Diamond, 1998). Now, in eLife, María C. Ávila-Arcos from Universidad Nacional Autónoma de México and colleagues – including Axel A Guzmán-Solís as first author – report the first likely evidence of viruses that originated in Africa circulating in Mexico during the colonial period (Guzmán-Solís et al., 2021).

The team (who are based in Mexico, Denmark and the United States) studied human remains that were found in two burial sites in Mexico City: one was associated with a colonial hospital and chapel, and the other a hospital that treated Native Americans during the 1576 Cocoliztli outbreak. Archaeological analysis of the remains from the latter site suggested that 21 of the individuals studied were of West African ancestry, despite their presence in a hospital associated with the treatment of indigenous people. Viral DNA was then extracted from these remains and analysed using various genetic sequencing tools.

The data identified an ancient strain of parvovirus B19 in three of the individuals studied, and an ancient strain of hepatitis B in one other individual. The genome of the discovered parvovirus was highly similar to a strain called genotype 3, which is sporadically found in Africa and is extremely rare in modern Europe and the Americas. This suggests that the transatlantic slave trade imposed by European colonization may have introduced new viral pathogens or genotypes into colonial Mexico.

Guzmán-Solís et al. also found the genotype 3 strain in the human remains of an indigenous person, suggesting that the virus may have spread to local individuals and contributed to the disease outbreaks that affected millions of people. The researchers then carried out genealogical DNA testing of the four individuals to study whether being infected with a specific pathogen genotype was associated with their genetic ancestry. These data helps build on a previous study revealing how different viral genotypes are distributed among modern Native Americans with African ancestry related to European colonial history (Lopera et al., 2014).

Although the study provides an important glimpse into the existence of these ancient viral strains in the colonial Americas, there are some limitations. First, the historical global distribution of the genotypes discovered is still largely unknown. Second, the conclusions are based on pathogens isolated from only four individuals, although it is common for these kinds of studies to have small amounts of genetic data due to limited number of samples.

Nevertheless, a global dataset with more genotype 3 sequences needs to be retrieved and analysed to confirm or refute the hypothesis that B19 genotype 3 was first brought to the Americas by enslaved Africans during the transatlantic slave trade. Indeed, a previous study found this strain of B19 in the remains of North European soldiers from the Second World War (Toppinen et al., 2015), and the genotype has also been observed in India (Jain et al., 2015) and the Brazilian Amazon (Freitas et al., 2008). Lastly, very few viral strains of African origin have been isolated from modern or ancient remains of African individuals, which will be needed to fully understand the genetic history and genotype distribution of these human infecting pathogens (Figure 1).

**Figure 1.**
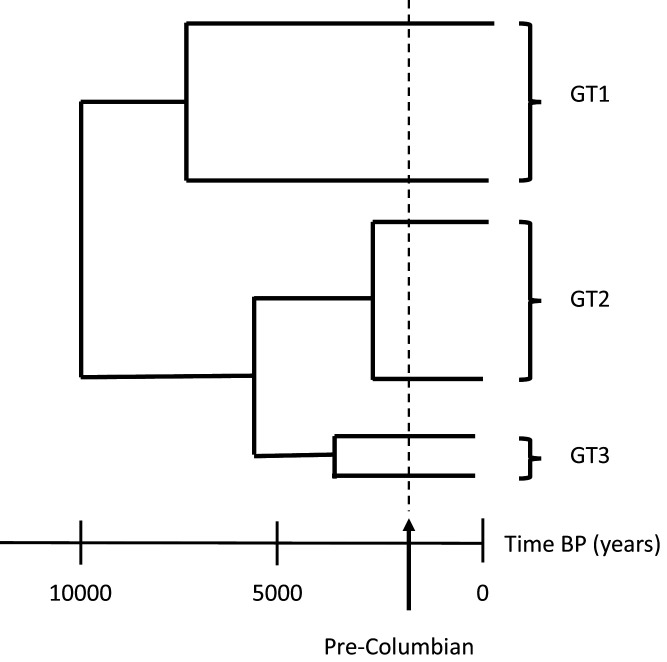
The evolution of parvovirus B19. In modern society, there are three main genotypes of parvovirus B19: genotype 1 (GT1), the most predominant strain around the world; genotype 2 (GT2) which is more commonly found in Europe; and genotype 3 (GT3) which has been sporadically observed worldwide, including in Ghana, Brazil and India (Jain et al., 2015; Freitas et al., 2008; Candotti et al., 2004). Previous studies have shown that the three genotypes likely emerged in human populations around five thousand years before present day (BP) from a common ancestor that arose ten thousand years ago. This divergence happened before the arrival of Columbus in the Americas (dashed line) and therefore pre-dates the European colonisation of the Americas and subsequent transatlantic slave trade.

Image credit: Ville N Pimenoff (based on data from Mühlemann et al., 2018).

Previous studies have found other ancient pathogens in the human remains of individuals who were alive during the colonial period in Mexico, some of whom were buried in a manner associated with an epidemic event. The work by Guzmán-Solís et al. suggests that parvovirus B19 can be added to this growing list of microbes, which includes the viruses and bacteria responsible for the skin disease yaws, hepatitis B, syphilis, salmonella and paratyphoid fever (Barquera et al., 2020; Schuenemann et al., 2018; Vågene et al., 2018). The study also provides historical evidence of viruses being transmitted between geographically distant human populations, which likely led to the emergence of related infectious diseases in populations that were previously naïve to the pathogen.
